# Higher urinary bisphenol A concentration and excessive iodine intake are associated with nodular goiter and papillary thyroid carcinoma

**DOI:** 10.1042/BSR20170678

**Published:** 2017-07-27

**Authors:** Zhenzhen Zhou, Jing Zhang, Fang Jiang, Yan Xie, Xiaochen Zhang, Ling Jiang

**Affiliations:** 1 Department of Endocrinology, Qilu Hospital of Shandong University, Jinan 250012, China; 2 Department of Pediatrics, Qilu Hospital of Shandong University, Jinan 250012, China; 3 Department of Islet Transplantation, Tianjin First Center Hospital, Tianjin 30000, China; 4 Heze Medical College, Heze 274000, China

**Keywords:** Bisphenol A, endocrine disrupting chemical, iodine excess intake, nodular goiter, papillary thyroid carcinoma

## Abstract

In the present study, we investigated whether bisphenol A (BPA) levels and excessive iodine intake were associated with papillary thyroid carcinoma (PTC) and nodular goiter (NG). We determined total BPA concentrations (TBC) in paired serum and urine samples, and urinary iodine concentrations (UIC) in urine samples collected from PTC patients, NG patients, and healthy individuals, then compared BPA concentrations and UIC within and between each patient group. The results showed that there were no gender-specific differences in serum TBC and UIC in each group, and no differences across all patient groups. Urinary BPA concentrations (UBC) were higher in the NG and PTC groups compared with the control group. UBC showed gender-specific differences in the NG and PTC group. Furthermore, UIC were higher in the NG and PTC groups compared with the control group. Higher UBC and excessive iodine intake were risk factors for NG and PTC according to multivariate logistic regression analysis. There was a significant correlation between UBC and UIC in each group. These data suggested that higher UBC and excessive iodine intake are associated with NG and PTC. The metabolic and functional pathways between BPA and iodine are potentially linked to the pathogenesis and progression of NG and PTC.

## Introduction

In recent years, studies have shown that thyroid-related diseases, such as nodular goiter (NG) and papillary thyroid carcinoma (PTC), are associated with endogenous estrogen activity [1–5].

Bisphenol A (BPA) is a widely used organic compound and applied in a variety of manufacturing processes [6]. Humans are exposed to BPA in a variety of ways, including dietary ingestion, dermal contact, inhalation, and intravenous administration [6–8]. BPA has shown to be detectable in human serum, urine, breast milk, cord blood, and mammary tissue [9]. Indeed, it has been detected in 92.6% of volunteers participating in the 2003–2004 National Health and Nutrition Examination in U.S.A. [10], and more than 80% in a sample of 129 Danish children and adolescents [11], and 50% of Chinese people [12]. Exposure to BPA is, therefore, highly prevalent worldwide. BPA is an endocrine disrupting chemical (EDC), acting as a ligand at the estrogen receptor, thereby influencing hormone biosynthesis and metabolism, and interfering with reproduction [13–16]. Studies have shown that free BPA can competitively bind to thyroid hormone receptors and inhibit the expression of genes regulated by thyroid hormones [17]. Few studies have examined the possible relationship between BPA and thyroid diseases, such as NG and PTC.

Iodine is an essential element for thyroid function and acquired from the diet. Deficiency or excess of iodine intake has been associated with thyroid disease, including autoimmune thyroiditis, hyperthyroidism, NG, and thyroid cancer [18–24]. However, the relationship between BPA exposure and excess iodine intake and their association with PTC and NG remain unclear.

In the present study, we examined serum and urine BPA levels and urine iodine levels in patients with NG and PTC, and investigated the relationship between BPA and iodine exposure and their potential association with NG and PTC.

## Materials and methods

### Patients and sample collection

Our study was approved by the committee on Human Research at Qilu Hospital of Shandong University, China. Written informed consent was provided by all participants in the study.

All participants were classified into three groups: PTC group, NG group, and healthy control group. Seventy-one patients with NG and 66 patients with PTC pathologically diagnosed were selected from February 2013 to September 2013 in Qilu Hospital of Shandong University, Jinan, Shandong Province, China. Patients were excluded according to the following criteria: (i) abnormal thyroid function, (ii) history of hyperthyroidism or hypothyroidism, (iii) administration of anti-thyroid drugs or thyroid hormone previously, and (iv) abnormal renal function or hepatic function. Finally, 53 PTC patients and 60 NG patients were included in the study. For the healthy control group, 148 healthy volunteers in Jinan were recruited and subjected to examination of the thyroid gland with thyroid ultrasound, and serum tests for thyroid function and hepatic/renal function. Those who had NGs, thyroid cysts, abnormal thyroid function, or abnormal hepatic or renal function were excluded. Finally, 65 volunteers were included into the healthy control group. Blood and spot urine samples were collected in the morning pre-operatively (fasting time >8 h). Serum samples were collected in 5 ml of BPA-free glass tubes from whole blood samples by centrifugation within 2 h, then stored at –80°C for further analysis. Urine samples were collected in 5 ml of BPA-free glass tubes and stored at –20°C. Thyroid hormones (free-T3, free thyroxine, and TSH) were determined with an autoanalyzer (ADVIA centaur Automated Chemiluminescence System, Simens AG, Germany) in Qilu Hospital. The measurement of urinary creatinine was performed with an autoanalyzer (Roche C8000 Chemistry System, Roche, Switzerland).

### Determination of BPA concentrations

Total (free plus conjugated) urinary and serum BPA concentrations were determined using an HPLC–MS/MS (TSQ vantage, Thermo Electron Corporation, U.S.A.) according to a previously published method [25] after zymohydrolysis by isotope-dilution in solid-phase extraction at the Shandong Province Analysis and Test Center, Shandong Academy of Sciences. In brief, 20 μl (50 ng) of D16-BPA (Dr Ehrenstorfer GmbH, Augsburg, Germany) and 50 μl of β-glucuronidase/sulfatase (Helixpomatia, Sigma–Aldrich, St. Louis, MO, U.S.A.) were dissolved in sodium acetate (pH 5.5), were added to urinary samples (1 ml) or serum samples (0.5 ml), mixed, diluted with 1 ml of water for urine and 2 ml of water for serum, and incubated in a water bath at 37°C for 3 h. Mixtures were then vacuum-pumped to pass through the 4 ml of methanol and 3 ml of water pre-conditioned C18 SPE (solid-phase extractor) cartridges (2.8 μm, 100 A, 2.1×100 mm, Agela Technologies Inc., Delaware, U.S.A.) at 1 ml/min and washed with 2 ml of water and 3 ml of water/methanol (3:20). BPA on the SPE cartridges was eluted with 4 ml of methanol into glass tubes, evaporated/dried in nitrogen, dissolved in 200 ml of methanol, and subjected to HPLC–MS/MS analysis. One to ten nanograms of BPA (Helixpomatia, Aldrich–Sigma, St. Louis, MO, U.S.A.) was spiked to the urine samples, and 5 ng of BPA was spiked to the serum of six healthy individuals as technical controls. The recovery of spiked BPA in the urinary samples ranged from 103 ± 7% to 99 ± 7% and the recovery of BPA was 98 ± 5% for serum samples. The relative standard deviation (RSD) of the analysis was <11%. Systematic error was analyzed using 1 ml of Mill-Q water spiked with 1 ng of BPA. The blank control of BPA in human urine or serum [26] was prepared by mixing samples from six healthy individuals. The linear range was 0.10–100 ng/ml for BPA, and the regression coefficient *R*^2^ was >0.995. The RSD were <10.8% and <8.6% for urine or serum samples respectively. SPE analysis was followed according to a previously published report [25]. The limit of quantification (LOQ) of BPA was 0.1 ng/ml for urine and 0.2 ng/ml for serum.

### Determination of iodine

UIC were determined according to the Sandell–Kolthoff reaction after ammonium persulfate treatment. The detection limit was 3 μg/l. The linear range of the standard curve was 0–300 μg/l with a standard deviation of 2.8–5.5%. The regression coefficient was *R*^2^ >99%. The recovery of iodine was 92.6–107.0%. According to the epidemiological criteria for assessing iodine nutrition based on median UIC announced by WHO and UNICEF, UIC <99 μg/l is considered as iodine deficiency, 100–199 μg/l as adequate iodine nutrition, 200–299 μg/l as above requirements of iodine intake, and >300 μg/l as excess iodine intake. In the present study, the UIC ranged from 142.90 to 1409.90 μg/l, so we classified the subjects into non-excessive iodine intake and excessive iodine intake according to the aforementioned UIC classifications in order to investigate the association between excessive iodine intake and thyroid diseases.

### Determination of creatinine and adjustment of BPA and iodine concentrations to creatinine

The concentration of creatinine in urine samples was determined using the basic picric acid method and used to normalize urine BPA and iodine concentrations (herein referred to as adjusted concentration) to eliminate variations resulting from sample processing and handling.

### Statistical analysis

Statistical analysis was performed using SPSS 19.0 software (SPSS Inc., Chicago, IL, U.S.A.). A value of 0.05 ng/ml was assigned as the standardized BPA level, which was below LOQ according to a previously published study [12,13]. Normal distribution of data, including creatinine-adjusted and unadjusted UIC, urinary BPA, and serum BPA concentrations, were tested using one-way analysis of variance. Other data were analyzed using Mann–Whitney *U* test. Analysis of Spearman’s rank correlation coefficient was performed to examine the relationship among paired serum and urinary BPA concentrations (UBC), creatinine-unadjusted/adjusted UBC and UIC. The Chi-square test was used to analyze the differences in the rates of excessive iodine intake and detection rates of UBC among the NG, PTC, and control groups. There was no standard range and classification of UBC. In order to analyze conveniently, we used receiver operating characteristic curve to cut off the UBC (>2.84 ng/ml, AUC = 0.70) and creatinine-adjusted UBC (>5.90 μg/g, AUC = 0.72). We carried out logistic regression analyses to evaluate the odds ratios (ORs) of the higher UBC (>2.84 ng/ml), creatinine-adjusted UBC (>5.90 μg/g), and excess iodine intake for thyroid disease.

## Results

### Urine but not serum BPA concentrations are associated with NG and PTC

We first examined BPA concentrations in all paired urine and serum samples. The results showed that all serum samples contained detectable BPA (free BPA and conjugated BPA) that ranged from 4.03 to 13.82 ng/ml (Table 1). There were no differences in serum TBC among the NG, PTC, and the healthy control groups (Table 1). UBC were detected in all samples of three groups (Table 1). UBC, either unadjusted or adjusted according to creatinine concentrations, in the NG group and the PTC group were significantly higher than those of the healthy control group (*P*=0.00 and *P*<0.04 respectively) (Table 1). However, there was no difference in the creatinine-unadjusted/adjusted UBC between the NG group and the PTC group (Table 1). These results suggested that high UBC but not serum TBC were associated with NG and PTC.

**Table 1:** Serum and UBC in the study groups

Characteristic	Overall	PTC group	NTG group	Healthy control group
**Participants**	178	53	60	65
**Sex**				
** Male**	50	14	14	22
** Female**	128	39	46	43
**Serum BPA (ng/ml)**				
** Detection rate (*N* / percent)**	178/100	53/100	60/100	65/100
** Range**	4.03–13.82	4.33–13.82	4.26–11.51	4.03–13.81
** GM**	7.42	7.61	7.07	7.62
** Median**	7.50	7.45	7.07	8.06
** P25**	6.61	6.87	6.28	4.70
** P75**	8.77	8.37	8.41	10.38
**Higher urinary BPA (>2.84 ng/ml) (*N* / percent)**	121/68	41/77	50/83	30/46
**Urinary BPA (ng/ml)**				
** Detection rate (*N* / percent)**	148/83	51/96	52/87	45/69
** Range**	0.05–34.46	0.05–34.46	0.05–30.67	0.05–11.52
** GM**	2.26	4.06^*^	3.35^*^	0.98
** Median**	4.18	4.66	5.03	2.56
** P25**	1.74	2.90	3.63	0.05
** P75**	7.01	7.68	8.43	5.40
**Urinary BPA/creatinine (μg/g)**				
** Range**	0.016–50.78	0.032–29.30	0.022–50.78	0.015–27.88
** GM**	2.82	4.68^*^	5.22^*^	1.06
** Median**	5.46	6.25	7.57	3.00
** P25**	2.18	3.01	3.71	0.05
** P75**	9.52	8.88	16.45	5.89

^*^P<0.05, there is statistical difference compared with the healthy control group; GM, geometric mean.

### Association of UIC with NG and PTC

We next examined UIC, which ranged from 142.90 to 1409.90 μg/l (Table 2). The results showed that the creatinine-unadjusted/adjusted UIC in the NG group and the PTC group were significantly higher than those of the control group (*P*=0.00 and *P*<0.04, respectively) (Table 2). However, there was no difference in UIC between the NG group and PTC group (Table 2). These results suggested that high UIC were associated with both NG and PTC.

**Table 2:** Iodine concentrations in all groups

Characteristic	Overall	PTC group	NTG group	Healthy control group
**Excessive iodine intake (*N* / percent)**	51/29	23/43*	22/37*	6/9
**Non-excessive iodine intake (*N* / percent)**	127/71	30/57	38/63	59/91
**Urinary iodine (ng/ml)**				
** Range**	142.90–1409.90	169.52–1409.90	144.10–1007.60	142.90–433.70
** GM**	268.25	319.66^*^	273.34^*^	228.51
** Median**	252.45	289.60	271.30	230.20
** P25**	215.33	240.20	198.30	184.20
** P75**	325.52	399.10	357.83	266.60
**Creatinine (g/l)**				
** Range**	0.10–3.24	0.20–2.66	0.11–2.24	0.10–2.24
** GM**	0.80	0.87	0.64	0.64
** Median**	0.85	0.94	0.70	0.70
** P25**	0.53	0.58	0.43	0.56
** P75**	1.35	1.31	1.07	1.59
**Urinary iodine/creatinine (μg/g)**				
** Range**	71.25–2995.74	94.85–2149.31	77.86–2995.74	71.25–2130.37
** GM**	335.05	368.71^*^	426.02^*^	248.26
** Median**	316.69	355.72	386.25	230.94
** P25**	193.57	194.63	260.34	148.57
** P75**	551.65	632.14	658.60	396.84

^*^P<0.05, there is statistical difference compared with the healthy control group.

We next examined the prevalence of excessive iodine intake (UIC >300 μg/l) and non-excessive iodine intake (UIC <300 μg/l) in the PTC group, NG group, and the healthy control group. The results showed that 43% of patients in the PTC group and 37% of patients in the NG group had excessive iodine intake, significantly higher than the control group, at only 9% (Table 2). This supported the notion that high UIC were associated with both NG and PTC. There was no significant difference in the prevalence of excessive iodine intake between the PTC group and the NG group, according to Chi-squared test (Table 2).

### Comparison of gender-specific BPA concentrations among the PTC, NG, and healthy control groups

We examined and compared gender-specific BPA concentrations among the PTC, NG, and healthy control groups. The results showed that there was no significant difference in serum TBC between males and females in each group, and indeed, across all groups (Table 3). Only unadjusted UBC in PTC group and adjusted UBC in NG group showed gender-specific differences. The unadjusted UBC in the male PTC group were significantly higher than the female PTC group (*P* =0.02). The adjusted UBC in the male NG group were significantly lower than the female NG group (*P*=0.01) (Table 3).

**Table 3:** Comparison of gender-specific BPA and iodine concentrations among the PTC, NG, and healthy control groups

Variables	GM	Median	P25	P75	Range
**PTC group**					
**Serum BPA (ng/ml)**					
** Male**	8.21	7.52	6.96	9.39	6.80–13.82
** Female**	7.40	7.40	6.64	8.13	4.33–12.67
**Unadjusted urinary BPA (ng/ml)**					
** Male**	7.27^‡^^,^*	6.35	4.43	12.62	1.99–34.46
** Female**	3.29^‡^^,^*	3.91	2.64	5.94	0.05–23.90
**Adjusted urinary BPA (μg/g)**					
** Male**	5.87*	6.75	2.98	9.09	2.11–27.38
** Female**	4.32*^,^^†^	6.25	2.86	8.90	0.03–29.30
**Unadjusted urinary iodine (ng/ml)**					
** Male**	346.68*	288.30	251.50	417.83	217.20–1409.90
** Female**	310.48*	289.60	235.90	401.70	169.52–1058.61
**Adjusted urinary iodine (μg/g)**					
** Male**	279.97	280.72	131.02	508.20	94.85–1120.23
** Female**	407.01*	414.92	196.03	637.08	111.11–2149.31
**NG group**					
**Serum BPA (μg/l)**					
** Male**	7.13	7.29	6.05	8.54	4.39–11.26
** Female**	7.05	7.07	6.30	8.35	4.26–11.51
**Unadjusted urinary BPA (ng/ml)**					
** Male**	3.24	4.56	3.75	5.70	0.05–7.97
** Female**	3.38*	5.32	3.60	9.25	0.05–30.67
**Adjusted urinary BPA (μg/g)**					
** Male**	3.45^‡^	3.99	3.12	7.54	0.10–11.34
** Female**	5.92^‡^^,^*^,^^†^	10.50	4.72	19.66	0.02–50.78
**Unadjusted urinary iodine (ng/ml)**					
** Male**	319.30*	288.30	252.14	402.00	153.10–1007.60
** Female**	260.71	246.83	190.10	341.20	144.10–813.25
**Adjusted urinary iodine (μg/g)**					
** Male**	339.20*	336.18	232.79	514.34	166.23–709.38
** Female**	456.62*	397.05	266.42	714.61	77.86–2995.74
**Healthy control group**					
**Serum BPA (ng/ml)**					
** Male**	8.18	8.37	7.33	9.62	4.53–13.52
** Female**	7.35	7.82	4.49	10.90	4.03–13.81
**Unadjusted urinary BPA (ng/ml)**					
** Male**	1.02	2.70	0.05	6.51	0.05–11.40
** Female**	0.96	2.39	0.05	4.57	0.05–11.52
**Adjusted urinary BPA (μg/g)**					
** Male**	0.96	2.64	0.03	7.06	0.02–18.08
** Female**	1.12	3.45	0.06	5.65	0.02–27.88
**Unadjusted urinary iodine (ng/ml)**					
** Male**	222.44	229.00	181.34	254.53	176.21–313.70
** Female**	231.68	230.50	185.30	268.27	142.90–433.70
**Adjusted urinary iodine (μg/g)**					
** Male**	209.88	162.17	134.40	391.02	82.38–658.62
** Female**	270.53	246.96	162.67	443.16	71.25–2130.37

^*^P<0.05, there is a statistical difference compared to the same gender in the healthy control group.

^†^P<0.05, there is a statistical difference in the same genders between the NG and PTC groups.

^‡^*P*<0.05, there is a statistical difference between the males and females in the PTC, NG, and control groups.

The unadjusted/adjusted UBC in the female NG group were significantly higher than those of the female control group (*P*=0.00/0.00) (Table 3). In addition, there was no significant difference in the unadjusted/adjusted UBC between the male NG group and the male control group.

The unadjusted/adjusted UBC in both the male PTC group (*P*=0.01/0.04) and the female PTC group (*P*=0.01/0.00) were significantly higher than those in the control group of the same gender (Table 3).

Adjusted UBC were significantly lower in the female PTC group than the female NG group (*P*=0.02), while there was no significant difference in unadjusted UBC between the female PTC group and the female NG group (*P*=0.09) (Table 3). There were no differences in unadjusted/adjusted UBC between males in the PTC and NG groups (Table 3).

### Comparison of gender-specific iodine concentrations among PTC, NG, and healthy control groups

We examined and compared gender-specific UIC in all three groups. The results showed that there were no significant differences in the unadjusted/adjusted UIC between males and females in each group, or between the PTC group and the NG group of the same gender (Table 3). Higher unadjusted/adjusted UIC were found in the PTC or NG groups, compared with the healthy control group of the same gender. Specifically, unadjusted/adjusted UIC were significantly higher in the male NG group than the male healthy control group (*P*=0.00/0.01) (Table 3), while adjusted UIC in the female NG group were significantly higher than the female healthy control group (*P*=0.00) (Table 3). Unadjusted/adjusted UIC in the female PTC group were higher than the female healthy control group (*P*=0.00/0.02) (Table 3). However, unadjusted UIC were higher in the male PTC group than the male healthy control group (*P*=0.00).

### Multivariate logistic regression analysis

We performed a multiple logistic analysis to examine the risk factors for NG and PTC by including age, gender, serum BPA concentration, urine creatinine, unadjusted and adjusted higher UBC, excessive iodine (unadjusted UIC), and adjusted UIC in the model. Our findings demonstrated that unadjusted higher UBC, excessive iodine intake, and age but not SBC or urine creatinine or adjusted UBC or adjusted UIC or gender were risk factors for both NG and PTC (Table 4).

**Table 4:** Analysis of risk factors for the PTC and NG using multivariable-adjusted logistic regression

Group	*B* (partial regression coefficient)	Crude OR	Adjusted OR	95% CI	*P*
**PTC group**					
Higher urinary BPA^*^ (>2.84 ng/ml)	1.27	3.99	3.57	1.37–9.30	0.01
Excess iodine intake^*^	1.72	7.54	5.61	1.84–17.07	0.00
Age	0.09	1.09	1.09	1.05–1.13	0.00
Gender	0.91	/	2.47	0.89–6.86	0.08
**NG group**					
Higher urinary BPA^*^ (>2.84 ng/ml)	1.82	5.83	6.15	2.16–17.53	0.00
Excess iodine intake^*^	1.32	5.69	4.93	1.17–12.10	0.03
Age	0.12	1.11	1.12	1.08–1.17	0.00
Gender	1.31	1.68	3.72	1.27–10.89	0.02

^*^Unadjusted urinary concentration.

### Correlation between serum and urinary BPA and iodine concentrations in PTC and NG patients

We performed a Spearman correlation analysis to examine the relationship between serum and urinary BPA and iodine concentrations in the PTC, NG, and healthy control groups. The results showed that no correlation was observed between serum and UBC, serum BPA and urinary iodine concentrations (UIC), unadjusted BPA and iodine concentrations in each group, although a significant correlation between the adjusted BPA and iodine concentrations in the PTC, NG, and healthy control groups was apparent (Table 5).

**Table 5:** Correlation between the urinary BPA and iodine concentrations in PTC and NTG patients

Group	*R*	*P*
**PTC (unadjusted)**	0.19	0.17
**PTC (adjusted)**	0.46	0.00
**NG (unadjusted)**	0.24	0.07
**NG (adjusted)**	0.49	0.00
**Control (unadjusted)**	−0.06	0.66
**Control (adjusted)**	0.53	0.00

## Discussion

In the present study, we measured the total BPA (free and conjugated) concentrations in all participants with a concentration of 7.42 ng/ml (geometry median value, GM). The detection rate was consistent with a Canadian study [27], in which free serum BPA concentrations were detected between 1.3 and 8.17 ng/ml in non-pregnant women. Free BPA in normal infants reached GM 1.70 ng/ml [28]. Considering that free BPA constitutes 20% of total serum BPA concentration, free BPA in our study subjects was GM 1.48 ng/ml, slightly lower than those in previous studies. In our study, urinary BPA was detected in 83% of participants with GM of 2.26 ng/ml. The results was consistent with the study by Zhang et al. [7], which reported urinary BPA was detectable in 84% of the Chinese adults with GM 1.01 ng/ml. In a U.S. study, urinary BPA was detectable in 93% of the participants with GM 2.6 ng/ml [29]. However, the GM of urinary BPA was 1.05 ng/ml for combined overt and subclinical hyperthyroidism and 0.63 ng/ml for combined overt and subclinical hypothyroidism patients [13]. In a Canadian population, urinary BPA was detectable in more than 90% of children and young adults, with a mean concentration of 1.3 μg/l [30]. Similar findings were demonstrated in a German population [31]. Workers constantly exposed to BPA in the BPA manufacturing factories in China have been shown to have a very high urinary BPA concentration with a median 84.6 mg/g creatinine [32]. In our study, the median adjusted urinary BPA concentration was 5.46 μg/g among study subjects. This suggests that serum and UBC may differ dramatically in different populations, geographical regions, and occupations.

We examined serum and urine BPA levels and urine iodine levels in patients with NG and PTC and found that high UIC and high UBC, but not serum TBC, were associated with NG and PTC. Unadjusted higher UBC, excessive iodine intake, and age, but not adjusted UBC or gender, were risk factors for the development of both NG and PTC. There was significant correlation between adjusted BPA and iodine concentrations, but not serum BPA and urinary iodine in the PTC, NG, and healthy control groups. These data supported the hypothesis that higher UBC and excessive iodine intake correlate and associate with PTC and NG.

In the present study, we found that BPA was detectable in all analyzed samples and no significant difference in serum TBC between males and females in each group and across PTC, NG, and healthy control groups was demonstrated, suggesting that all groups were equally exposed to BPA. This is consistent with a previous study carried out in children and pregnant women in different age groups in China [7]. Free BPA (20%), BPA disulfate (34%), and BPA glucuronide (46%) levels were found in the participants of this study [25]. Absorbed BPA is metabolized in the liver by conjugation with glucuronide and rapidly excreted in the urine within 24 h [33]. BPA entering the bloodstream is eliminated via excretion in the urine [33]. Different from serum BPA, urine BPA comprises of free BPA (32%), BPA disulfate (7%), BPA glucuronide (57%), and BPA chlorides (4%) [25]. Further, urine BPA levels relative to creatinine levels were calculated in order to eliminate errors in determining distribution of BPA. In contrast with a previous study [7], we found no association between serum and UBC.

In the present study, we found that UBC, either unadjusted or adjusted according to creatinine concentration, in the NG group and the PTC group were significantly higher than those of the healthy control group. Multivariate logistic regression analysis also showed that unadjusted higher UBC was a risk factor for both NG and PTC. Therefore, high UBC, but not serum TBC, are likely to be associated with NG and PTC. We further examined and compared gender-specific UBC in all three groups. In consistent with the overall UBC in the NG and PTC groups, UBC in the female NG group and both the male and female PTC groups are higher than those in the control group of the same gender. Adjusted UBC were significantly lower in the female PTC group than the female NG group. However, we found that there was no significant difference in unadjusted/adjusted UBC between the male NG group and the male control group, or between males in the PTC and NG groups. This gender-specific association has also been demonstrated in several studies examined in different populations [7,10]. Furthermore, unadjusted UBC in the male PTC group were higher than those in females, which is consistent with another study from China [12]. These findings suggest that higher BPA levels may pose a more significant impact on women than men with NG and PTC, as supported by the notion that BPA is estrogenic, as well as an EDC [34–36].

In the present study, we found that the adjusted UBC were significantly lower in the female PTC group than the female NG group and there was no difference in serum BPA levels, suggesting higher renal elimination of BPA in female NG groups than females in the PTC group. Absorbed BPA was metabolized in the liver by conjugation with glucuronide and excreted through urine within 24 h [33]. There was more free BPA (32% versus 20%), less BPA disulfate (7% versus 34%), BPA glucuronide (57% versus 46%), and BPA chlorides (4%) in urine than serum [25]. BPA is a potential substrate for the efflux transporters multidrug resistance-associated proteins (MRP2 and MRP3), and breast cancer-resistant protein (BCRP), and BPA glucuronide would likely enter the systemic and portal blood supply through basolateral MRP3 [37]. Whether renal expression of these transporters is differential or gender-specific and would play a differential role in renal excretion of BPA and its metabolites in female NG groups remains to be investigated in the future.

It has been established that a local reduction in iodine content in follicular lumen leads to overexpression of local thyroid-stimulating hormone receptor, which in turn excessively stimulates the regional thyroid tissue, and results in the formation of NG. Paradoxically, we found that UIC were higher in NG and PTC groups than the healthy control group, and all UIC were above 100 μg/l, which indicated no iodine deficiency in all participants, according to WHO and UNICEF guidelines [38]. Multivariate logistic regression analysis also showed that excessive iodine intake was a risk factor for both NG and PTC. This is consistent with studies in patients with PTC, with or without lymph node metastasis [39], and in patients with thyroid cancer or benign nodules [40]. Notably, studies have shown that long-term high iodine intake reduced the expression of Na^+^/I^−^ symporter (NIS) gene and protein and induced oxidative stress in rats [41,42]. High iodine intake increased the occurrence of T1799 BRAF mutation (69% versus 53%), which was a risk factor for PTC [43]. PTC patients with a BRAF mutation had lower NIS mRNA expression levels [44]. This suggests that high iodine results in changes in the genes such as NIS and BRAF, which may in turn cause local deficiency of iodine in the follicular lumen, as well as proliferation and heteroplasia in the thyroid gland, leading to PTC and NG. There were no gender-specific findings with respect to UIC in NG and PTC, as revealed in our present study.

Interestingly, we found that there was a significant correlation between adjusted BPA and iodine concentrations in the PTC, NG, and healthy control groups. Studies have shown that BPA increases estrogen-related receptor γ (ERRγ) mRNA and protein levels in breast cancer cells [45]. An inverse agonist of ERRγ, GSK5182, increases iodine uptake and enhances membrane localization of NIS in anaplastic thyroid cancer cells [46]. TSH also up-regulates NIS mRNA and protein levels both *in vitro* and *in vivo* [47]. Urinary BPA negatively relates to thyroid-stimulating hormone levels in adults [13]. The maternal BPA was also inversely associated with TSH in newborn girls [48]. However, in another study, urinary BPA and serum TSH had a positive association [49]. Therefore, we speculate that there was a cross-talk in the metabolic and functional pathways between BPA and iodine in PTC and NG patients.

There are several limitations to the findings in our study. First, we cannot estimate the causal relationships in the present study, as it was a cross-sectional study. Second, we only evaluated single spot urine samples, and did not determine time-course changes in urinary BPA and iodine concentrations. Third, we measured total BPA but did not measure free BPA concentrations. Free BPA is the most toxic form of BPA to humans. Fourth, our sample size was limited, and information on dietary habits, geographical location, or socioeconomic status was not incorporated. Fifth, the age of the control group was younger than the PTC and NG groups, instead of equal ages in all groups, although equal exposure of BPA in all groups was revealed by measuring serum BPA concentrations. We will address these limitations in future studies.

In conclusion, we demonstrated that UBC and UIC were higher in NG and PTC groups compared with healthy controls. Higher UBC and excessive iodine intake were risk factors for NG and PTC development. Association of high UBC and UIC with NG and PTC is gender-dependent. There may, therefore, be a potential cross-talk in the metabolic and functional pathways between BPA and iodine in the pathogenesis and progression of NG and PTC.

## Abbreviations

BPA: bisphenol A
EDC: endocrine disrupting chemical
ERRγ: estrogen-related receptor γ
LOQ: limit of quantification
NG: nodular goiter
PTC: papillary thyroid carcinoma
RSD: relative standard deviation
TBC: total BPA concentrations
UBC: urinary BPA concentrations
AUC: area under the curve
UIC: urinary iodine concentrations
